# Association between cumulative uric acid to high-density lipoprotein cholesterol ratio and the incidence and progression of chronic kidney disease

**DOI:** 10.3389/fendo.2023.1269580

**Published:** 2023-12-14

**Authors:** Peipei Liu, Junjuan Li, Ling Yang, Zihao Zhang, Hua Zhao, Naihui Zhao, Wenli Ou, Yinggen Zhang, Shuohua Chen, Guodong Wang, Xiaofu Zhang, Shouling Wu, Xiuhong Yang

**Affiliations:** ^1^ School of Public Health, North China University of Science and Technology, Tangshan, Hebei, China; ^2^ Department of Nephrology, Kailuan General Hospital, Tangshan, Hebei, China; ^3^ Hebei Key Laboratory for Chronic Diseases, Tangshan Key Laboratory for Preclinical and Basic Research on Chronic Diseases, School of Basic Medical Sciences, North China University of Science and Technology, Tangshan, Hebei, China; ^4^ Department of Nuclear Medicine, Kailuan General Hospital, Tangshan, Hebei, China; ^5^ Department of Cardiology, Kailuan General Hospital, Tangshan, Hebei, China

**Keywords:** chronic kidney disease, progression, uric acid to high-density lipoprotein cholesterol ratio, cumulative exposure, UHR

## Abstract

**Objective:**

The ratio of uric acid to high-density lipoprotein cholesterol (UHR) was related to the risk of chronic kidney disease (CKD), we aimed to investigate the association of cumulative UHR (cumUHR) with incidence and progression of CKD.

**Methods:**

Our study included a total of 49,913 participants (mean age 52.57 years, 77% males) from the Kailuan Study conducted between 2006 and 2018. Participants who completed three consecutive physical examinations were included. Cumulative UHR (cumUHR) was computed as the summed average UHR between two consecutive physical examinations, multiplied by the time between the two examinations. Participants were then categorized into four groups based on cumUHR quartiles. Subsequently, participants were further divided into a CKD group and a non-CKD group. The associations between cumUHR and CKD and it’s progression were assessed by Cox proportional hazards regression models. The cumulative incidence of endpoint events was compared between the cumUHR groups using the log-rank test. The C-index, net reclassification improvement (NRI) and integrated discrimination improvement (IDI) were calculated to assess the predictive performance of cumUHR.

**Results:**

After a mean follow-up of 8.0 ± 1.7 years, there were 4843 cases of new-onset CKD, 2504 of low eGFR, and 2617 of proteinuria in the non-CKD group. Within the CKD group, there were 1952 cases of decline in eGFR category, 1465 of >30% decline in eGFR, and 2100 of increased proteinuria. In the non-CKD group, the adjusted hazard ratios (HRs) and confidence intervals (CIs) in the fourth quartile were 1.484 (1.362–1.617), 1.643 (1.457–1.852), and 1.324 (1.179–1.486) for new-onset CKD, low eGFR, and proteinuria, respectively. In the CKD group, the adjusted HRs in the fourth quartile were 1.337 (1.164–1.534), 1.428 (1.216–1.677), and 1.446 (1.267–1.651) for decline in eGFR category, >30% decline in eGFR, and increase in proteinuria, respectively. In addition, we separately added a single UHR measurement and cumUHR to the CKD base prediction model and the CKD progression base prediction model, and found that the models added cumUHR had the highest predictive value.

**Conclusion:**

High cumUHR exposure was an independent risk factor for the incidence and progression of CKD, and it was a better predictor than a single UHR measurement.

## Introduction

Chronic kidney disease (CKD) has emerged as a growing global health concern, with alarming statistics highlighting its escalating prevalence and associated mortality. Between 1990 and 2017, the global incidence of CKD surged by 29.3%, accompanied by a notable 41.5% increase in mortality rates. In 2017 alone, CKD affected approximately 9.1% of the world’s population, impacting an estimated 700 million individuals, with 132 million cases reported in China (1). This surge in prevalence, coupled with the resulting mortality rates and soaring medical expenses, has placed substantial burdens on healthcare systems globally, particularly in countries like China. Recognizing the imperative to address this health crisis, pinpointing risk factors for CKD assumes paramount significance. Notably, the identification of biomarkers, such as the ratio of uric acid to high-density lipoprotein cholesterol (UHR), holds promise in surpassing the efficacy of traditional risk factors like diabetes, hypertension, dyslipidemia, and obesity. Unraveling these potential biomarkers could revolutionize early detection strategies, facilitating the identification of high-risk populations and enabling proactive prevention and intervention measures (1–6).

The uric acid (UA) to high-density lipoprotein cholesterol (HDL-C) ratio (UHR) serves as a valuable biomarker, offering insights into chronic inflammation and metabolic status (7). Previous research has linked an elevated UHR to heightened risks of type 2 diabetes (8), metabolic syndrome (9), and thyroiditis (10). Notably, a recent cross-sectional study revealed a positive correlation between elevated UHR and the risk of CKD (6). Despite these findings, there is a notable gap in our understanding regarding the association between UHR and the incidence and progression of CKD.

To bridge this gap, our focus centers on cumulative UHR (cumUHR) exposure, derived from repeated measurements that provide a more comprehensive reflection of both exposure duration and intensity when compared to baseline UHR. Our objective is to explore the association between cumUHR and CKD as well as its progression within the Kailuan Study cohort (study registration number: ChiCTR-TNC-11001489).

## Methods

### Study population

The study population was derived from the ongoing prospective Kailuan Study, initiated in 2006 with follow-up visits at 2-year intervals. Baseline and follow-up examinations include measurements of blood UA, HDL-C, serum creatinine, and urinary protein. To assess the association between cumUHR and CKD, we selected individuals who participated in the baseline examination and completed two consecutive follow-up visits as our study population. The inclusion criteria were as follows: participation in the Kailuan Group health examinations in 2006, 2008, and 2010; complete data on UA, HDL-C, serum creatinine, and urinary protein for all three examinations; and written informed consent obtained before study enrollment. Exclusion criteria encompassed individuals with an estimated glomerular filtration rate (eGFR) <15 mL/min/1.73 m^2^ or heavy proteinuria (urinary protein ≥3+) and those with missing follow-up serum creatinine or urinary protein data were excluded. The study was approved by the Ethics Committee of Kailuan General Hospital and adhered to the principles of the Declaration of Helsinki.

### Data collection

The methods used to collect epidemiological, anthropometric, and biochemical indicators in the Kailuan Study have been described elsewhere (11). Height, body weight, blood pressure, and related measurements are obtained by trained medical staff using standardized methods.

On the day of examination, 5 mL of venous blood are collected from each participant between 07:00 and 09:00 after fasting for more than 8 h for determination of serum UA, HDL-C, creatinine, and urinary protein. Biochemical parameters are measured using an automatic biochemical analyzer (Hitachi 7600; Tokyo, Japan) in the central laboratory at Kailuan General Hospital. Serum UA is measured using an enzymatic method, HDL-C using a direct test method (Mind Bioengineering Co., Ltd., Shanghai, China), and creatinine using an enzymatic method with an intra-batch coefficient of variation of <10%, an inter-batch relative range of <10%, and a linear range of 44–106 μmol/L. The specific methods used to detect other biochemical indicators in blood have been published previously (12).

Urinalysis was performed using a dry chemistry method and a urine sediment detection method (H12-MA urine analysis reagent strips and DIRUI N-600 urine routine detection analyzer sourced from Changchun Derui Medical Technology Co., Ltd.). Urinary protein was measured using the semi-quantitative test strip method [(-), <15 mg/dL; trace, 15–29 mg/dL; (1+), 30–300 mg/dL; (2+), 300–1000 mg/dL; 3+), >1000 mg/dL].

### Calculations and grouping

#### Definition and calculation of UHR and cumUHR

UHR was defined as the ratio of uric acid and high-density lipoprotein cholesterol.


UHR=UA/HDL−C

CumUHR was defined as the sum of the average UHR values recorded on two consecutive physical examinations multiplied by the time interval between the two examinations (13, 14) as follows:


$$\text{CumUHR}=\left[\left(\text{UH}{\text{R}}_{2006}+\text{UH}{\text{R}}_{2008}\right)/2{\;}^{*}\text{tim}{\text{e}}_{1-2}\right]\;+\;\left[\left(\text{UH}{\text{R}}_{2008}\;+\text{UH}{\text{R}}_{2010}\right)/2{\;}^{*}\text{tim}{\text{e}}_{2-3}\right]$$

where UHR_2006_, UHR_2008_, and UHR_2010_ represent the UHR measured during the physical examinations in 2006, 2008, and 2010, respectively. Time_1-2_ and time_2-3_ represent the specific time intervals between consecutive physical examinations.

The participants were then divided according to their cumUHR value into quartiles: first cumUHR quartile, <906.64; second cumUHR quartile, ≥906.64 to <1089.70; third cumUHR quartile, ≥1089.70 to <1419.25; and fourth cumUHR quartile, ≥1419.25.

#### Calculation of eGFR

eGFR was calculated using the CKD-EPI (Chronic Kidney Disease Epidemiology Collaboration) formula (15). In women, eGFR = 144 × (sCr/0.7)^-0.329^ × 0.993^Age^ if sCr ≤0.7 mg/dL and 144 × (sCr/0.7)^-1.209^ × 0.993^Age^ if sCr is >0.7 mg/dL. In men, eGFR = 144 × (sCr/0.9)^-0.411^ × 0.993^Age^ if sCr is ≤0.9 mg/dL and 144 × (sCr/0.9)^-1.209^ × 0.993^Age^ if sCr is >0.9 mg/dL.

### Definitions

According to the latest clinical practice guidelines from KDIGO (Kidney Disease Improving Global Outcomes) (16), CKD is defined as eGFR <60 mL/min/1.73 m^2^ and/or urine protein positive (17), low eGFR is defined as eGFR <60 mL/min/1.73 m^2^, and proteinuria is defined as a semi-quantitative urinary protein value ≥1+. According to the KDIGO guidelines (18) and the recent report by the CKD Prognosis Consortium (19), CKD progression is defined as (1) a decline in eGFR category (≥90 [G1], 60–89 [G2], 45–59 [G3a], 30–44 [G3b], 15–29 [G4], and <15 [G5] mL/min/1.73 m^2^) accompanied by a decrease in eGFR of ≥25% from baseline), (2) a >30% decline in eGFR from baseline, or (3) an increase in proteinuria (i.e., an increase in semi-quantitative urine protein [–, ±, 1+, 2+, 3+]) (20).

Hypertension is defined as a systolic blood pressure ≥140 mmHg, a diastolic blood pressure ≥90 mmHg, or current use of antihypertensive medication. Diabetes is defined as fasting blood glucose ≥7.0 mmol/L or use of antidiabetic medication. Alcohol consumption is defined as an average daily intake of 100 mL of liquor (alcohol content ≥50%) for at least 1 year. Smoking is defined as smoking at least 1 cigarette per day on average in the past year. Physical exercise is defined as exercising ≥3 times per week, with each session lasting ≥30 minutes. Body mass index (BMI) is calculated as weight (kg) divided by the square of height (m).

### Follow-up times and determination of endpoint events

The baseline for follow-up of study participants was completion of the 2010 health examination. For participants without CKD, the endpoint events included new-onset CKD, low eGFR, and proteinuria, with the follow-up endpoint being the last health examination (December 31, 2019). In the case of participants with CKD, the endpoint events were defined as a decline in eGFR category, a >30% decline in eGFR, and an increase in proteinuria, and the follow-up endpoint was the last health examination (December 31, 2019). In instances where a participant experienced two or more endpoint events, the earliest time and event were utilized as the outcome.

### Statistical analysis

The baseline data for this study were collected from the 2010 health examination. Normally distributed continuous variables are expressed as the mean ± standard deviation, and group comparisons were conducted using one-way analysis of variance. For non-normally distributed continuous variables, the median (interquartile range) was utilized, and non-parametric (Kruskal-Wallis) tests were employed for between-group comparisons. Categorical variables are expressed as percentages, and group comparisons were assessed using chi-squared tests. Participants were stratified into cumUHR quartiles, and a Cox proportional hazards regression model was used to analyze the association between cumUHR levels and incidence and progression of CKD. Models were adjusted progressively for age, sex, educational level, smoking, alcohol consumption, physical exercise, BMI, low-density lipoprotein cholesterol, high-sensitivity C-reactive protein, baseline eGFR (in 2010), hypertension, diabetes, antihypertensive, antidiabetic medications, and the UHR level in 2006. The cumulative incidence rate of endpoint events in different cumUHR groups was calculated using the Kaplan-Meier method, and group comparisons were performed using the log-rank test. The CKD prediction model by Nelson et al. (21) served as the CKD base model, and a CKD progression prediction model was developed based on traditional risk factors, including age, sex, and systolic blood pressure as the CKD progression base model (22), UHR_2006_ and cumUHR were respectively added to calculate the C-index (C-statistic), net reclassification improvement (NRI), and integrated discrimination improvement (IDI) for comparing the predictive abilities of UHR2006 and cumUHR for the incidence and progression of CKD.

To further confirm the robustness of our study, we used a Cox model stratified by time intervals and fitted Cox models with time-varying coefficients. All analyses were performed using SAS version 9.4 (SAS Institute Inc., Cary, NC, USA). All p-values were two-tailed and considered statistically significant at <0.05.

## Results

### General characteristics of participants according to cumUHR group

**Figure 1:**
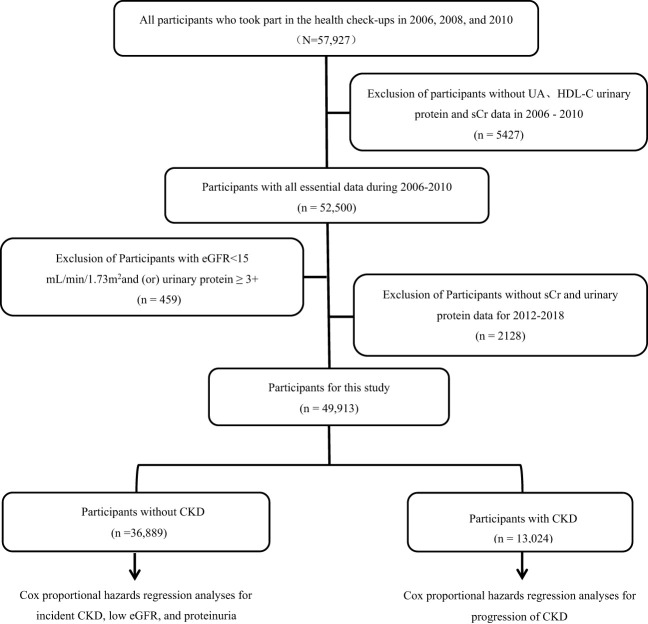
Flow chart showing the process used to select study participants. CKD, chronic kidney disease; eGFR, estimated glomerular filtration rate; HDL-C, high-density lipoprotein cholesterol; sCr, serum creatinine; UA, uric acid.

A total of 57,927 participants underwent physical examinations in 2006, 2008, and 2010, and they were included in the study. After excluding participants with missing UA, HDL-C, urine protein, or serum creatinine data from the three physical examinations (n=542), those with an eGFR <15 mL/min/1.73 m^2^ and/or heavy proteinuria (n=45), and those with missing follow-up urine protein and serum creatinine data (n=2128), 49,913 participants (mean age of 52.57 ± 11.77 years, 77.0% males) were available for analysis (**Figure 1**). Participants were divided into four quartiles based on cumUHR level: first quartile, 746.34 ± 115.21; second quartile, 1004.72 ± 63.55; third quartile, 1273.68 ± 95.52; and fourth quartile, 1870.00 ± 593.72. Increasing cumUHR was associated with gradual increases in participant age, BMI, high-sensitivity C-reactive protein, systolic blood pressure, fasting blood glucose, and UA levels and a decrease in the HDL-C level. There were also gradual increases in the proportions of men, smokers, consumers of alcohol, and those who exercised regularly, as well as in the proportions of participants who tested positive for urine protein, had hypertension or diabetes, or were taking antihypertensive or antidiabetic medication. The differences between the groups were statistically significant (P<0.05, **Table 1**). Of the 49,913 participants, 13,024 (26.09%) had CKD, and 36,889 (73.91%) did not. The baseline characteristics of the non-CKD and CKD groups according to cumUHR quartile are shown in **Supplementary Tables 1**, **2**, respectively.

**Table 1:** Baseline characteristics of all study participants.

Variable	Total (N=49,913)	Quartile 1 (n=12,478)	Quartile 2 (n=12,478)	Quartile 3 (n=12,478)	Quartile 4 (n=12,479)	*P -value
Age, years	52.57 ± 11.77	51.08 ± 10.67	51.52 ± 11.51	53.18 ± 11.89	54.52 ± 12.60	<0.01
Male sex, n (%)	38417 (77.0)	7512 (60.2)	9349 (74.9)	10268 (82.3)	11288 (90.4)	<0.01
Education level, n (%)						<0.01
Lower than university or college	36006 (72.1)	9231 (74.0)	8984 (72.0)	9080 (72.8)	8711 (69.8)	
University or college or higher	13908 (27.9)	3247 (26.0)	3494 (28.0)	3399 (27.2)	3768 (30.2)	
Current smoker, n (%)	20262 (40.6)	3771 (30.2)	4568 (36.6)	5568 (44.6)	6355 (50.9)	<0.01
Current drinker, n (%)	21643 (43.4)	4149 (33.2)	4788 (38.4)	5919 (47.4)	6787 (54.4)	<0.01
Physical activity, n (%)	7260 (14.5)	1353 (10.8)	1418 (11.4)	1949 (15.6)	2540 (20.4)	<0.01
Body mass index	25.14 ± 3.36	24.07 ± 3.25	24.77 ± 3.23	25.42 ± 3.27	26.29 ± 3.29	<0.01
LDL-C, mmol/L	2.59 ± 0.82	2.59 ± 0.83	2.60 ± 0.76	2.60 ± 0.85	2.57 ± 0.82	<0.01
hs-CRP, mg/L	1.09 (0.50–2.57)	0.90 (0.40–2.23)	0.89 (0.25–2.20)	1.23 (0.60–2.89)	1.30 (0.70–2.84)	<0.01
eGFR, mL/min/1.73 m^2^	90.50 ± 19.78	91.81 ± 19.81	88.94 ± 20.35	91.10 ± 19.16	90.13 ± 19.69	<0.01
SBP, mmHg	130.61 ± 19.01	127.81 ± 18.68	129.37 ± 18.39	131.61 ± 18.99	133.64 ± 19.77	<0.01
FBG, mmol/L	5.65 ± 1.51	5.60 ± 1.58	5.62 ± 1.49	5.68 ± 1.51	5.70 ± 1.41	<0.01
UA, µmol/L	294.86 ± 89.09	230.44 ± 58.00	265.33 ± 66.44	314.62 ± 74.19	369.03 ± 86.93	<0.01
HDL-C, mmol/L	1.54 ± 0.47	1.75 ± 0.50	1.57 ± 0.45	1.49 ± 0.43	1.34 ± 0.39	<0.01
Antidiabetic medication, n (%)	2852 (5.71)	629 (5.04)	610 (4.89)	794 (6.36)	819 (6.56)	<0.01
Antihypertensive medication, n (%)	5760 (11.5)	859 (6.88)	1008 (8.08)	1516 (12.1)	2377 (19.0)	<0.01
Diabetes, n (%)	7257 (14.5)	1726 (13.8)	1788 (14.3)	1841 (14.8)	1902 (15.2)	<0.01
Hypertension, n (%)	22260 (44.6)	4560 (36.5)	5104 (40.9)	5852 (46.9)	6744 (54.0)	<0.01
cumUHR	1221.59 ± 42.16	746.34 ± 115.21	1004.72 ± 63.55	1273.68 ± 95.52	1870.00 ± 593.72	<0.01
UHR_2006_	197.93 ± 93.36	130.85 ± 37.90	168.62 ± 43.37	207.36 ± 52.93	284.89 ± 125.45	<0.01
UHR_2010_	209.28 ± 95.76	139.09 ± 46.77	178.60 ± 57.67	224.46 ± 73.50	294.97 ± 111.00	<0.01

*P, comparison of baseline characteristics according to cumulative UHR quartile.

cumUHR, cumulative uric acid to high-density lipoprotein cholesterol ratio; eGFR, estimated glomerular filtration rate; FBG, fasting blood glucose; HDL-C, high-density lipoprotein cholesterol; hs–CRP, high–sensitivity C-reactive protein; LDL-C, low–density lipoprotein cholesterol; SBP, systolic blood pressure; TC, total cholesterol; UA, uric acid; UHR_2006,_ uric acid to high-density lipoprotein cholesterol ratio in 2006.

### Incidence and progression of CKD according to cumUHR group

**Figure 2:**
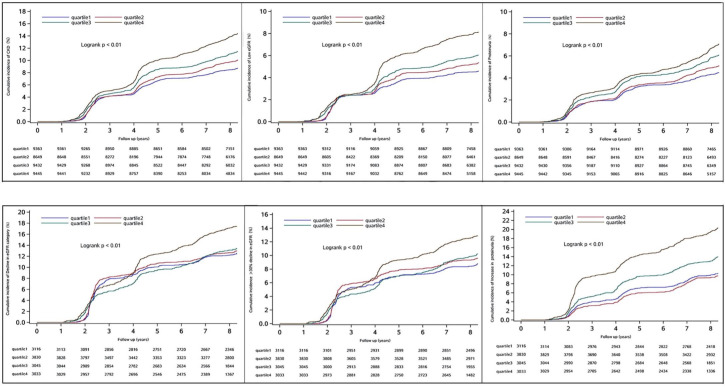
Cumulative incidences for new-onset chronic kidney disease, low eGFR, proteinuria, decline in eGFR category, >30% decline in eGFR, and increase in proteinuria. CKD, chronic kidney disease; eGFR, estimated glomerular filtration rate.

Over a mean follow-up of 8.0 ± 1.7 years, the non-CKD group experienced 4843 cases of new-onset CKD, 2504 instances of low eGFR, and 2617 cases of proteinuria. In the CKD group, with a mean follow-up of 8.0 ± 1.6 years, there were 1952 cases of a decline in eGFR category, 1465 cases of >30% decline in eGFR, and 2100 cases of increased proteinuria. The corresponding cumulative incidence rates across various cumUHR groups are presented in **Figure 2** (log-rank test P < 0.01).

### Multivariate Cox regression analysis of cumUHR exposure and incidence and progression of CKD

In the non-CKD group, utilizing cumUHR quartile as the independent variable and CKD, low eGFR, and proteinuria as dependent variables, with the first quartile as the reference group, the multivariate-adjusted HRs for new-onset CKD in the second to fourth quartiles were 1.12 (95% CI 1.03–1.22), 1.22 (95% CI 1.12–1.33), and 1.48 (95% CI 1.36–1.62), respectively (P_trend_<0.01); the corresponding HRs for developing low eGFR were 1.16 (95% CI 1.12–1.43), 1.27 (95% CI 1.12–1.43), and 1.64 (95% CI 1.46–1.85), while those for developing proteinuria were 1.06 (95% CI 0.94–1.19), 1.19 (95% CI 1.06–1.33), and 1.32 (95% CI 1.18–1.49) (P_trend_<0.01). For each 1 standard deviation increase in cumUHR, the HRs for developing CKD, low eGFR, and proteinuria were 1.09 (95% CI 1.07–1.11), 1.09 (95% CI 1.07–1.11), and 1.08 (95% CI 1.06–1.11), respectively (**Table 2**).

**Table 2:** Hazard ratios for new-onset CKD, low eGFR, and proteinuria in study participants without CKD according to CumUHR quartile.

	Quartile 1 HR (95% CI)	Quartile 2 HR (95% CI)	Quartile 3 HR (95% CI)	Quartile 4 HR (95% CI)	Per SD	*P* for trend
Participants, n	9363	8649	9432	9445		
CKD						
Cases, n (%)	1022 (10.92)	1052 (12.16)	1274 (13.51)	1495 (15.83)		
Incidence rate (per 1000 person-years)	13.40	15.11	17.06	20.65		
Model 1	Reference	1.13 (1.03–1.23)	1.26 (1.15–1.37)	1.53 (1.41–1.67)		
Model 2	Reference	1.12 (1.03–1.22)	1.22 (1.12–1.33)	1.48 (1.36–1.62)	1.09 (1.07–1.11)	<0.01
Model 3	Reference	1.11 (1.02–1.21)	1.22 (1.12–1.34)	1.49 (1.35–1.66)		
Low eGFR						
Cases, n (%)	495 (5.29)	538 (6.22)	646 (6.85)	825 (8.73)		
Incidence rate (per 1000 person-years)	6.35	7.55	8.41	11.05		
Model 1	Reference	1.18 (1.05–1.34)	1.29 (1.14–1.45)	1.70 (1.51–1.91)		
Model 2	Reference	1.16 (1.12–1.43)	1.27 (1.12–1.43)	1.64 (1.46–1.85)	1.09 (1.07–1.11)	<0.01
Model 3	Reference	1.16 (1.02–1.31)	1.26 (1.11–1.43)	1.61 (1.40–1.86)		
Proteinuria						
Cases, n (%)	575 (6.14)	566 (6.54)	709 (7.52)	767 (8.12)		
Incidence rate (per 1000 person-years)	7.36	7.91	9.22	10.20		
Model 1	Reference	1.08 (0.96–1.21)	1.25 (1.11–1.40)	1.42 (1.26–1.59)		
Model 2	Reference	1.06 (0.94–1.19)	1.19 (1.06–1.33)	1.32 (1.18–1.49)	1.08 (1.06–1.11)	<0.01
Model 3	Reference	1.07 (0.95–1.20)	1.22 (1.08–1.37)	1.41 (1.22–1.62)		

CKD was defined as eGFR <60 mL/min/1.73 m^2^ and/or proteinuria. Low eGFR was defined as eGFR <60 mL/min/1.73 m^2^. Proteinuria was defined as urinary protein ≥1+ on dipstick testing.

Model 1 was adjusted for age and sex.

Model 2 was adjusted for age, sex, smoking, alcohol consumption, education level, physical activity, body mass index, low-density lipoprotein cholesterol, high-sensitivity C-reactive protein, diabetes, antidiabetic treatment, hypertension, and antihypertensive treatment.

Model 3 was adjusted for all the variables in model 2 and UHR_2006._

CI, confidence interval; CKD, chronic kidney disease; CumUHR, cumulative uric acid to high-density lipoprotein cholesterol ratio; eGFR, estimated glomerular filtration rate; HR, hazard ratio; SD, standard deviation; UHR_2006,_ uric acid to high-density lipoprotein cholesterol ratio in 2006.

In the CKD group, using cumUHR quartile as the independent variable and decline in eGFR category, >30% decline in eGFR, and increase in proteinuria as the dependent variables, with the first quartile of cumUHR as the reference group, the multivariate-adjusted HRs for decline in eGFR category in the second to fourth quartiles were 1.06 (95% CI 0.93–1.02), 1.07 (95% CI 0.93–1.22), and 1.34 (95% CI 1.16–1.53) (P_trend_<0.01); the respective HRs for >30% decline in eGFR were 1.12 (0.96–1.30), 1.17 (0.99–1.37), and 1.43 (1.22–1.68) (P_trend_<0.01); the HRs in the second to fourth quartiles for an increase in proteinuria were 0.96 (95% CI 0.85–1.10), 1.15 (95% CI 1.01–1.32), and 1.45 (95% CI 1.27–1.65) (P_trend_<0.01). For each 1 standard deviation increase in cumUHR, the HRs for decline in eGFR category, >30% decline in eGFR, and increase in proteinuria were 1.07 (95% CI 1.04–1.10), 1.04 (95% CI 1.02–1.07), and 1.06 (95% CI 1.04–1.08), respectively (**Table 3**).

**Table 3:** Hazard ratios for decline in eGFR and increase in proteinuria in study participants with CKD by CumUHR quartile.

	Quartile 1 HR (95% CI)	Quartile 2 HR (95% CI)	Quartile 3 HR (95% CI)	Quartile 4 HR (95% CI)	Per SD	P for trend
Participants, n	3117	3830	3045	3033		
Decline in eGFR category						
Cases, n (%)	426 (13.67)	533 (13.92)	442 (14.52)	551 (18.17)		
Incidence rate (per 1000 person-years)	17.57	18.03	18.98	24.81		
Model 1	Reference	1.07 (0.94–1.22)	1.14 (0.99–1.30)	1.51 (1.32–1.72)		
Model 2	Reference	1.06 (0.93–1.20)	1.07 (0.93–1.22)	1.34 (1.16–1.53)	1.07 (1.04–1.10)	<0.01
Model 3	Reference	1.05 (0.92–1.20)	1.06 (0.92–1.22)	1.32 (1.12–1.55)		
>30% decline in eGFR						
Cases, n (%)	304 (9.75)	396 (10.34)	341 (11.20)	424 (13.98)		
Incidence rate (per 1000 person-years)	12.09	12.95	14.14	18.18		
Model 1	Reference	1.14 (0.98–1.33)	1.27 (1.08–1.48)	1.67 (1.43–1.95)		
Model 2	Reference	1.12 (0.96–1.30)	1.17 (0.99–1.37)	1.43 (1.22–1.68)	1.04 (1.02–1.07)	<0.01
Model 3	Reference	1.11 (0.95–1.29)	1.15 (0.97–1.35)	1.38 (1.15–1.66)		
Increase in proteinuria						
Cases, n (%)	424 (13.60)	507 (13.24)	517 (16.98)	652 (21.50)		
Incidence rate (per 1000 person-years)	17.06	16.55	22.14	29.78		
Model 1	Reference	0.96 (0.85–1.11)	1.27 (1.11–1.45)	1.66 (1.46–1.89)		
Model 2	Reference	0.96 (0.85–1.10)	1.15 (1.01–1.32)	1.45 (1.27–1.65)	1.06 (1.04–1.08)	<0.01
Model 3	Reference	0.96 (0.85–1.10)	1.16 (1.01–1.33)	1.45 (1.24–1.70)		

Decline in eGFR category was defined as a certain decrease in eGFR category accompanied by a ≥25% decrease in eGFR from baseline. A 30% decline in eGFR was defined as a >30% decrease in eGFR from baseline. An increase in proteinuria was defined as a certain increase in semiquantitative urinary protein (–, ±, 1+, 2+, 3+) on dipstick testing.

Model 1: adjusted for age and sex.

Model 2 was adjusted for age, sex, smoking, alcohol consumption, education level, physical activity, body mass index, low-density lipoprotein cholesterol, high-sensitivity C-reactive protein, diabetes, antidiabetic treatment, hypertension, and antihypertensive treatment.

Model 3 was adjusted for all the variables in model 2 and UHR_2006._

CI, confidence interval; CKD, chronic kidney disease; CumUHR, cumulative uric acid to high-density lipoprotein cholesterol ratio; eGFR, estimated glomerular filtration rate; HR, hazard ratio; SD, standard deviation; UHR_2006,_ uric acid to high-density lipoprotein cholesterol ratio in 2006.

To determine whether the effect of cumUHR on new-onset CKD and progression of CKD could be influenced by a single UHR measurement, we further adjusted for the 2006 UHR level, and the results were consistent with these reported above (**Tables 2**, **3**).

### Stratified analysis

We observed significant interactions between cumUHR and sex (P-interaction = 0.047) with risk of CKD. The association between cumUHR and CKD was more pronounced in female population (**Table S3**).

**Figure 3:**
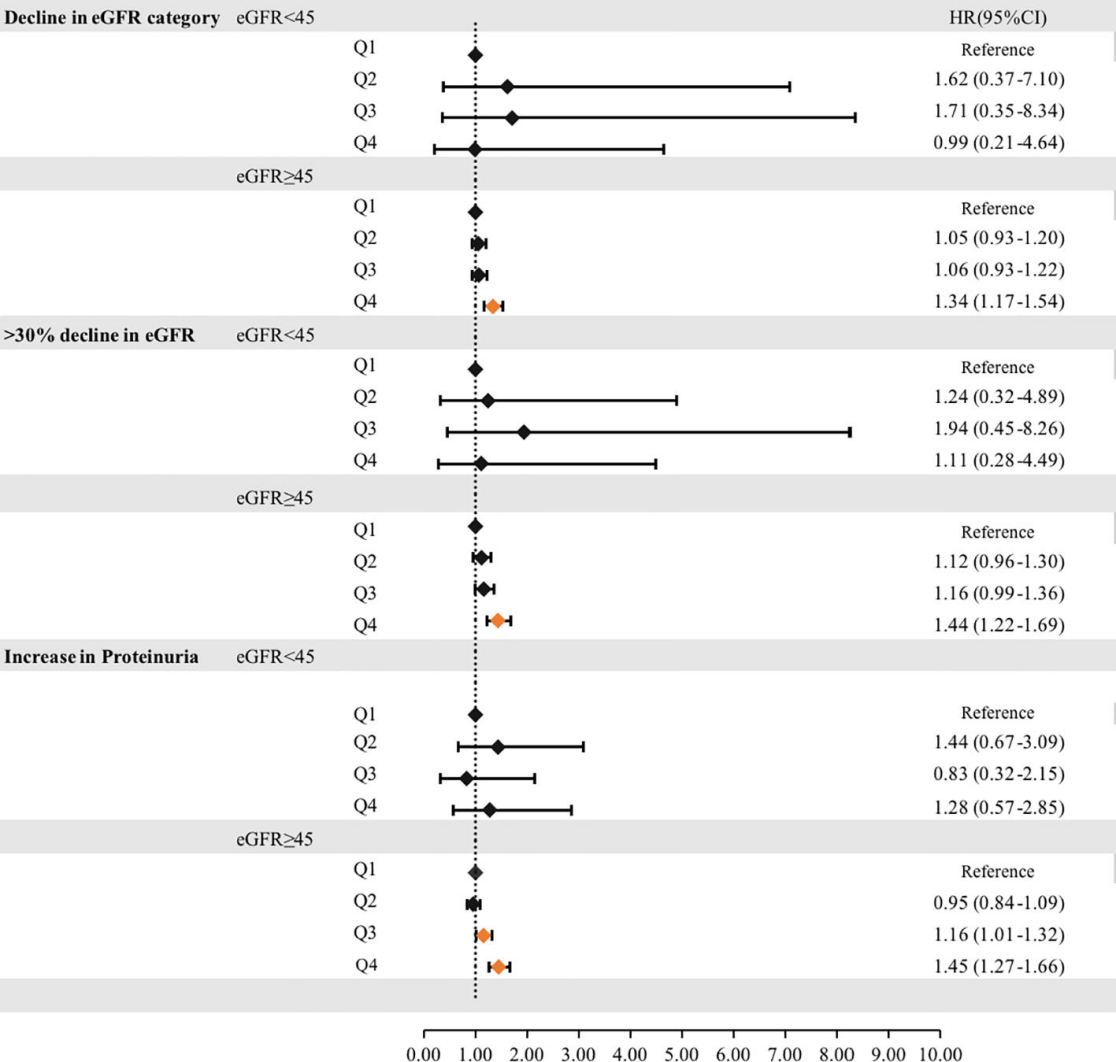
Hazard ratios for incident progression of chronic kidney disease according to the cumUHR quartile stratified by eGFR (≥45 mL/min/1.73 m^2^or <45 mL/min/1.73 m^2^) are shown. CI, confidence interval; cumUHR, cumulative uric acid to high-density lipoprotein cholesterol ratio; eGFR, estimated glomerular filtration rate; HR, hazard ratio; Q, quartile.

To reduce the impact of CKD on cumUHR, we further stratified the CKD population into subgroups according to whether eGFR was ≥45 or <45 mL/min/1.73 m^2^; after adjusting for confounding factors, the HRs for decline in eGFR category, >30% decline in eGFR, and increase in proteinuria were 1.34 (95% CI 1.17–1.54), 1.44 (95% CI 1.22–1.69), and 1.45 (95% CI 1.27–1.66) in the group with eGFR ≥45 mL/min/1.73 m^2^ group (P<0.01). Conversely, there were no associations in the group with eGFR <45 mL/min/1.73 m^2^ (P<0.01, **Figure 3**).

### C-index, NRI, and IDI

We separately added UHR_2006_ and cumUHR to the CKD base prediction model and the CKD progression base prediction model, and found that the cumUHR model had the highest predictive value. Compared with the UHR_2006_ model, the C-index of cumUHR model increased by 0.09%, 0.1% and 0.13% for new-onset CKD, low eGFR, and proteinuria, respectively, in the non-CKD group. The C-index of cumUHR model increased by 0.21%, 0.27% and 0.46% for decline in eGFR category, >30% decline in eGFR, and increase in proteinuria in the CKD group. In addition, compared with the base models, both NRI and IDI of cumUHR model were higher than those of the UHR2006 model (**Tables 4**, **5**).

**Table 4:** C-index, IDI, and NRI of the base model with and without cumUHR or UHR_2006_ for chronic kidney disease.

		C-index	IDI	P for IDI	NRI	*P* for NRI
CKD	Base model	0.6440	Reference	Reference	Reference	Reference
	Base + UHR_2006_	0.6454	0.0061	<0.01	0.0480	<0.01
	Base + cumUHR	0.6463	0.0132	<0.01	0.1040	<0.01
Low eGFR	Base model	0.7288	Reference	Reference	Reference	Reference
	Base + UHR_2006_	0.7312	0.0020	<0.01	0.1161	<0.01
	Base + cumUHR	0.7322	0.0033	<0.01	0.1482	<0.01
Proteinuria	Base model	0.6047	Reference	Reference	Reference	Reference
	Base + UHR_2006_	0.6050	0.00003	0.12	0.0161	0.44
	Base + cumUHR	0.6063	0.0022	<0.01	0.0690	<0.01

The base model included age, sex, estimated glomerular filtration rate, cardiovascular history, smoking, hypertension, body mass index, and urinary protein. Abbreviations: C-index, C-statistic; cumUHR, cumulative uric acid to high-density lipoprotein cholesterol ratio; NRI, net reclassification index; IDI, integrated discrimination improvement; UHR_2006,_ uric acid to high-density lipoprotein cholesterol ratio in 2006.

**Table 5:** C-index, IDI, and NRI of the base model with and without cumUHR or UHR_2006_ for progression of chronic kidney disease (stage G3a).

		C-index	IDI	P for IDI	NRI	P for NRI
Decline in eGFR category	Base model	0.6937	Reference	Reference	Reference	Reference
	Base + UHR_2006_	0.6922	0.0008	<0.01	0.0829	<0.01
	Base + cumUHR	0.6943	0.0019	<0.01	0.1203	<0.01
>30% decline in eGFR	Base model	0.6923	Reference	Reference	Reference	Reference
	Base + UHR_2006_	0.6901	0.0010	<0.01	0.1112	<0.01
	Base + cumUHR	0.6928	0.0020	<0.01	0.1466	<0.01
Increase in proteinuria	Base model	0.6229	Reference	Reference	Reference	Reference
	Base + UHR_2006_	0.6278	0.0007	<0.01	0.1091	<0.01
	Base + cumUHR	0.6324	0.0016	<0.01	0.1760	<0.01

The base model included age, sex, estimated glomerular filtration rate, urinary protein, systolic blood pressure, fasting blood glucose, and total cholesterol. Abbreviations: C-index, C-statistic; cumUHR, cumulative uric acid to high-density lipoprotein cholesterol ratio; NRI, net reclassification index; IDI, integrated discrimination improvement; UHR_2006,_ uric acid to high-density lipoprotein cholesterol ratio in 2006.

### Sensitivity analysis

The time-varying cox model stratified by time intervals and fitted with resampling methods also showed consistent results with the conventional Cox proportional model (**Tables S4**, **S5**).

## Discussion

In this study, we found that high cumUHR not only associated with the increased risk of new-onset CKD, low eGFR, and proteinuria in the population without CKD, but also associated with the increased risk of a decline in eGFR and an increase in proteinuria in the population with stage G3a CKD. The association between cumUHR and new-onset CKD was more pronounced in the female population. Furthermore, the association between cumUHR and CKD and progression of stage G3a CKD was independent of and superior to that of a single UHR measurement and also had a better effect on predictive ability.

After an average follow-up of 8.0 ± 1.7 years, we found that the risk of new-onset CKD, low eGFR, and proteinuria increased by 48.4%, 64.3%, and 32.4%, respectively, in the fourth cumUHR quartile in the non-CKD group and that the risk of decline in eGFR category, >30% decline in eGFR, and increase in proteinuria increased by 33.7%, 42.8%, and 44.6%, respectively, in the fourth cumUHR quartile in the CKD group. The risks of CKD and progression of CKD increased significantly with increasing cumUHR level. To our knowledge, this is the first prospective cohort study to report an association of a high cumUHR with an increased risk of new-onset CKD and progression of CKD. Previous cross-sectional studies found positive associations of the UHR with a decline in eGFR and risk of CKD in the general population aged around 50 years, with a 9.28-fold higher risk of CKD in the fourth quartile of UHR compared with that in the first quartile (6). Our study, which measured UA and HDL-C levels multiple times and calculated the cumUHR while considering the cumulative and time effects of exposure factors, provides more reliable results. Therefore, we consider cumUHR to be a new potential risk factor for CKD and progression of CKD that is independent of traditional factors.

Based on earlier cohort studies, cumulative exposure to risk factors such as blood pressure, blood lipids, C-reactive protein, UA, and the monocyte-to-HDL cholesterol ratio has been shown to have a greater impact on adverse outcomes such as cardiovascular disease than single measurements (13, 23–26). Similarly, our study demonstrates that the ability of cumUHR to predict CKD and progression of CKD is superior to that of a single UHR measurement. After adjusting for the UHR_2006_ measurement in the model, there was a slight increase in the HRs for cumUHR and the risk of development and progression of CKD. Furthermore, we incorporated UHR_2006_ and cumUHR into the CKD prediction models developed by Nelson et al. (21) and into our traditional risk factor-based CKD progression prediction model. The results showed that the predictive ability of both models was significantly improved by addition of cumUHR in comparison with UHR_2006_, indicating that the effect and predictive ability of cumUHR on CKD and progression of CKD are independent and superior to that of a single UHR measurement.

The results of this study provide evidence that cumUHR is a risk factor for new-onset CKD and progression of G3a stage CKD. UHR has the dual advantage of wide availability and cost-effectiveness in clinical Settings, and the use of electronic medical records makes CumUHR an easily accessible metric. Therefore, CumUHR can be used as a basis to identify individuals at high risk of CKD in the general population and individuals at high risk of progression in the G3a CKD population.

It is also worth noting that we found a stronger association between cumUHR exposure and risk of CKD in the female population. Previous studies have identified that older women have a higher risk of developing CKD than men when exposed to the same level of risk factors, such as hypertension and hyperlipidemia (27, 28). Therefore, women with a higher cumUHR should be monitored carefully for CKD. Furthermore, our stratified analysis of the CKD population found that the cumUHR was positively correlated with the risk of eGFR decline and increase in proteinuria in the population with an eGFR ≥45 mL/min/1.73 m^2^. The CRIC cohort study showed a positive correlation between the UA level and the risk of deterioration of kidney function (progression to dialysis or kidney transplantation) in the population with G2 or G3a stage CKD (29), which indirectly supports our observations. However, we did not find any association between cumUHR and progression of CKD in the population with an eGFR <45 mL/min/1.73 m^2^. The CRIC study did not find a correlation between UA and the risk of deterioration of kidney function in the G3b population but found a potential protective effect in the G4 stage population. The reasons for this phenomenon may include (1) the small size of the population with an eGFR <45 mL/min/1.73 m^2^ in our study (only 303 cases) and (2) residual confounding caused by severe renal failure or severe metabolic disorders, malnutrition, and other factors in patients on long-term dialysis. Therefore, further clinical randomized controlled trials and large-scale prospective cohort studies are needed to confirm the precise association between cumUHR and progression of CKD in the population with an eGFR <45 mL/min/1.73 m^2^.

Previous research indicates that an elevated serum uric acid (UA) level is an independent risk factor for the development of new-onset CKD or rapid decline in kidney function in Chinese health examination populations (30). Basic research has shown that UA is the final product of purine metabolism (degradation of adenine and guanine) (31). UA can promote intracellular oxidation, induce endothelial dysfunction, and cause renal fibrosis and glomerulosclerosis (32, 33). At the same time, hyperuricemia stimulates the renin–angiotensin system, leading to hypertension and renal vascular constriction, producing a vicious cycle that further exacerbates progression of kidney disease (34). Furthermore, HDL-C is a highly heterogeneous class of lipoproteins, the main members of which are ApoA1 and ApoA2, which have major antioxidant and anti-inflammatory properties that effectively prevent atherosclerosis (35). Low HDL-C is associated with an increased risk of inflammatory diseases (36). Additional evidence suggests that low HDL-C levels serve as an independent predictive indicator for the progression of chronic kidney disease (CKD) (37), and a decrease in HDL-C levels increases the risk of the occurrence and progression of CKD (38). There is also some evidence that low HDL-C increases the risk of hyperuricemia (39). Therefore, the impact of high cumUHR on the onset and progression of CKD may be influenced by the combined cross-effects of the above mechanisms; when both show reverse changes, the increase in risk is more significant. When screening high-risk populations, individuals with elevated UA and decreased HDL-C require careful attention, especially if female and CKD is stage G3a.

The results of this study provide evidence that cumUHR is a risk factor for new-onset CKD and progression of G3a stage CKD. UHR has the dual advantage of wide availability and cost-effectiveness in clinical Settings, and the use of electronic medical records makes CumUHR an easily accessible metric. Therefore, CumUHR can be used as a basis to identify individuals at high risk of CKD in the general population and individuals at high risk of progression in the G3a CKD population.

This study had a prospective cohort design and included a large sample size with long-term follow-up and repeated measurements of variables. Furthermore, measurement of cumulative exposure can reflect both exposure dose and exposure time, effectively avoiding potential regression dilution bias. However, the study also had some limitations. First, all study participants were employees of the Kailuan Group, with a higher proportion of men than women, which may have had an impact on our results. Second, urine protein was detected using a semi-quantitative strip test and 24-hour creatinine clearance was not measured, which may have led to diagnostic errors in CKD (40). However, other studies have shown that the semi-quantitative strip test has high sensitivity (93.3%) and specificity (91.6%) for diagnosis of CKD. Finally, due to the presence of missing data, 2128 participants were excluded from the analysis. Excluded participants may differ from those included in the study in certain aspects, which may lead to potential bias.

## Conclusion

This study demonstrates that high cumUHR exposure is an independent risk factor and predictor of the incidence of CKD and progression of stage G3a CKD. The ability of cumUHR to predict CKD and progression of G3a stage CKD is superior to that of a single UHR measurement.

## Data availability statement

The original contributions presented in the study are included in the article/**Supplementary Material**. Further inquiries can be directed to the corresponding authors.

## Ethics statement

The studies involving humans were approved by the Ethics Committee of Kailuan General Hospital (approval number:2006-05). The studies were conducted in accordance with the local legislation and institutional requirements. Written informed consent for participation in this study was provided by the participants’ legal guardians/next of kin.

## Author contributions

PL: Writing – original draft, Investigation, Writing – review & editing. JL: Writing – review & editing. LY: Formal Analysis, Writing – review & editing. ZZ: Formal analysis, Writing – review & editing. HZ: Formal Analysis, Writing – review & editing. NZ: Formal Analysis, Writing – review & editing. WO: Data curation, Writing – review & editing. YZ: Software, Writing – review & editing. SC: Data curation, Writing – review & editing. GW: Data curation, Writing – review & editing. XZ: Writing – review & editing. SW: Project administration, Resources, Writing – review & editing. XY: Funding acquisition, Project administration, Writing – review & editing.
