# Supplementary material for: Association between cumulative uric acid to high-density lipoprotein cholesterol ratio and the incidence and progression of chronic kidney disease
Source: Front Endocrinol (Lausanne). 2023 Dec 14;14:1269580. doi: 10.3389/fendo.2023.1269580 (PMC10753577; doi:10.3389/fendo.2023.1269580)
Supplement: Supplementary file 1 [file Table_1.docx]

**List of Supplementary Materials**

**Supplementary Table S1.** Baseline characteristics of study participants with chronic kidney disease

**Supplementary Table S2.** Baseline characteristics of study participants without chronic kidney disease

**Supplementary Table S3.** Hazard ratios for new-onset chronic kidney disease according to cumulative UHR quartile stratified by sex

**Supplementary Table S4.** Hazard ratios for new-onset CKD, low eGFR, and proteinuria in study participants without CKD according to CumUHR quartile (time-varying cox)

**Supplementary Table S5.** Hazard ratios for decline in eGFR and increase in proteinuria in study participants with CKD by CumUHR quartile (time-varying cox)

| **Supplementary Table S1.** Baseline characteristics of study participants with chronic kidney disease | | | | | | |
| --- | --- | --- | --- | --- | --- | --- |
| Variable | Total (N=13,024) | Quartile 1 (n=3116) | Quartile 2 (n=3830) | Quartile 3 (n=3045) | Quartile 4 (n=3033) | ***P *-*value |
| Age, years | 55.97 ± 12.20 | 54.15 ± 11.32 | 53.30 ± 11.76 | 56.83 ± 12.26 | 60.35 ± 12.24 | <0.01 |
| Male sex, n (%) | 9687 (74.4) | 1868 (59.9) | 2833 (74.0) | 2383 (78.3) | 2603 (85.8) | <0.01 |
| Education level, n (%) |  |  |  |  |  | <0.01 |
| Lower than university or college | 10030 (77.0) | 2464 (79.1) | 2881 (75.2) | 2370 (77.8) | 2315 (76.4) |  |
| University or college or higher | 2994 (23.0) | 653 (20.9) | 949 (24.8) | 675 (22.2) | 717 (23.6) |  |
| Current smoker, n (%) | 4441 (34.1) | 718 (23.0) | 1144 (29.9) | 1212 (39.8) | 1367 (45.1) | <0.01 |
| Current drinker, n (%) | 4441 (34.1) | 745 (23.9) | 1131 (29.5) | 1204 (39.5) | 1361 (44.9) | <0.01 |
| Physical activity, n (%) | 2420 (18.6) | 395 (12.7) | 464 (12.1) | 630 (20.7) | 931 (30.7) | <0.01 |
| Body mass index | 25.51 ± 3.44 | 24.54 ± 3.36 | 25.12 ± 3.28 | 25.82 ± 3.36 | 26.68 ± 3.40 | <0.01 |
| LDL-C, mmol/L | 2.58 ± 0.81 | 2.55 ± 0.80 | 2.56 ± 0.71 | 2.60 ± 0.88 | 2.62 ± 0.88 | <0.01 |
| hs–CRP, mg/L | 1.10 (0.20–2.84) | 0.77 (0.01–2.30) | 0.43 (0.01–2.00) | 1.50 (0.60–3.39) | 1.70 (0.90–3.60) | <0.01 |
| TC, mmol/L | 4.99 ± 0.97 | 5.02 ± 0.92 | 4.87 ± 0.87 | 5.03 ± 1.00 | 5.10 ± 1.10 | <0.01 |
| UA, µmol/L | 286.91 ± 90.89 | 226.56 ± 60.07 | 255.32 ± 65.92 | 310.74 ± 77.01 | 364.89 ± 93.15 | <0.01 |
| HDL-C, mmol/L | 1.45 ± 0.40 | 1.64 ± 0.43 | 1.46 ± 0.34 | 1.43 ± 0.39 | 1.29 ± 0.36 | <0.01 |
| SBP, mmHg | 134.84+20.49 | 132.13 ± 20.18 | 131.90 ± 19.50 | 136.66 ± 20.57 | 139.52 ± 20.88 | <0.01 |
| FBG, mmol/L | 5.79 ± 1.70 | 5.69 ± 1.75 | 5.70 ± 1.63 | 5.88 ± 1.80 | 5.90 ± 1.61 | <0.01 |
| eGFR, mL/min/1.73 m^2^ | 77.47 ± 21.01 | 77.79 ± 20.36 | 75.82 ± 20.67 | 78.86 ± 20.62 | 77.80 ± 22.30 | <0.01 |
| Antidiabetic treatment, n (%) | 1050 (8.06) | 221 (7.09) | 239 (6.24) | 280 (9.20) | 310 (10.2) | <0.01 |
| Antihypertensive treatment, n (%) | 1721 (13.2) | 216 (6.93) | 289 (7.55) | 454 (14.9) | 762 (25.1) | <0.01 |
| Diabetes, n (%) | 2558 (19.6) | 572 (18.4) | 697 (18.2) | 631 (20.7) | 658 (21.7) | <0.01 |
| Hypertension, N (%) | 6874 (52.8) | 1409 (45.2) | 1790 (46.7) | 1705 (56.0) | 1970 (65.0) | <0.01 |
| cumUHR | 1221.59 ± 542.16 | 762.89 ± 111.81 | 1001.72 ± 62.87 | 1269.49 ± 94.25 | 1922.55 ± 681.67 | <0.01 |
| UHR_2006_ | 192.13 ± 92.12 | 126.01 ± 35.91 | 162.71 ± 40.96 | 204.85 ± 54.78 | 284.48 ± 125.36 | <0.01 |
| *P, comparison of baseline characteristics according to cumulative UHR quartile. Abbreviations: cumUHR, cumulative uric acid to high-density lipoprotein cholesterol ratio; eGFR, estimated glomerular filtration rate; FBG, fasting blood glucose; HDL-C, high-density lipoprotein cholesterol; hs–CRP, high–sensitivity C-reactive protein; LDL-C, low–density lipoprotein cholesterol; SBP, systolic blood pressure; TC, total cholesterol; UA, uric acid; UHR_2006,_ uric acid to high-density lipoprotein cholesterol ratio in 2006. | | | | | | |

| **Supplementary Table S2.** Baseline characteristics of study participants without chronic kidney disease | | | | | | |
| --- | --- | --- | --- | --- | --- | --- |
| Variable | Total  (N=36,889) | Quartile 1  (n=9363) | Quartile 2  (n=8649) | Quartile 3  (n=9432) | Quartile 4  (n=9445) | ***P-value |
| Age, years | 51.37 ± 11.37 | 50.09 ± 10.25 | 50.71 ± 11.31 | 52.01 ± 11.53 | 52.59 ± 12.13 | <0.01 |
| Male sex, n (%) | 28730 (77.9) | 5644 (60.3) | 6516 (75.3) | 7885 (83.6) | 8685 (91.9) | <0.01 |
| Education level, n (%) |  |  |  |  |  | <0.01 |
| Lower than university or college | 25975 (70.4) | 6767 (72.3) | 6103 (70.5) | 6710 (71.1) | 6395 (67.7) |  |
| University or college or higher | 10923 (29.6) | 2596 (27.7) | 2548 (29.5) | 2726 (28.9) | 3053 (32.3) |  |
| Current smoker, n (%) | 15821 (42.9) | 3053 (32.6) | 3424 (39.6) | 4356 (46.2) | 4988 (52.8) | <0.01 |
| Current drinker, n (%) | 17202 (46.6) | 3404 (36.4) | 3657 (42.3) | 4715 (50.0) | 5426 (57.4) | <0.01 |
| Physical activity, n (%) | 4840 (13.1) | 958 (10.2) | 954 (11.0) | 1319 (14.0) | 1609 (17.0) | <0.01 |
| Body mass index | 25.01 ± 3.33 | 23.92 ± 3.20 | 24.61 ± 3.20 | 25.29 ± 3.24 | 26.17 ± 3.24 | <0.01 |
| LDL-C, mmol/L | 2.59 ± 0.82 | 2.61 ± 0.84 | 2.61 ± 0.79 | 2.60 ± 0.85 | 2.55 ± 0.80 | <0.01 |
| hs–CRP, mg/L | 1.09 (0.50–2.45) | 0.96 (0.42–2.21) | 1.00 (0.40–2.30) | 1.20 (0.60–2.68) | 1.20 (0.67–2.60) | <0.01 |
| eGFR, mL/min/1.73 m^2^ | 95.10 ± 17.11 | 96.48 ± 17.26 | 94.75 ± 17.27 | 95.05 ± 16.86 | 94.09 ± 16.97 | <0.01 |
| SBP, mmHg | 128.99 ± 18.24 | 126.31 ± 17.90 | 128.12 ± 17.65 | 129.91 ± 18.09 | 131.54 ± 18.84 | <0.01 |
| FBG, mmol/L | 5.59 ± 1.41 | 5.56 ± 1.51 | 5.57 ± 1.41 | 5.61 ± 1.38 | 5.63 ± 1.32 | <0.01 |
| UA, µmol/L | 297.51 ± 87.99 | 231.73 ± 57.24 | 269.80 ± 66.11 | 315.88 ± 73.05 | 369.74 ± 84.35 | <0.01 |
| HDL-C, mmol/L | 1.57 ± 0.49 | 1.79 ± 0.50 | 1.62 ± 0.49 | 1.51 ± 0.44 | 1.35 ± 0.40 | <0.01 |
| Antidiabetic treatment, n (%) | 1802 (4.88) | 409 (4.36) | 370 (4.28) | 515 (5.45) | 508 (5.39) | <0.01 |
| Antihypertensive treatment, n (%) | 4745 (12.9) | 1050 (11.2) | 1095 (12.7) | 1242 (13.2) | 1358 (14.4) | <0.01 |
| Diabetes, n (%) | 32199 (87.3) | 8217 (87.7) | 7561 (87.4) | 8233 (87.2) | 8188 (86.8) | <0.01 |
| Hypertension, n (%) | 16613 (45.0) | 3456 (36.9) | 3624 (41.9) | 4465 (47.3) | 5068 (53.7) | <0.01 |
| Cardiovascular history | 1566 (4.24） | 198 (0.54） | 288 (0.78） | 444 (1.20） | 636 (1.72） | <0.01 |
| cumUHR | 1224.44+509.75 | 741.00+115.85 | 1006.31 ± 63.82 | 1275.53+96.04 | 1853.89+561.84 | <0.01 |
| UHR_2006_ | 199.98+93.71 | 132.47+38.40 | 171.28+44.15 | 208.19 ± 52.28 | 285.17+125.54 | <0.01 |
| *P, comparison of baseline characteristics according to cumulative UHR quartile. Abbreviations: cumUHR, cumulative uric acid to high-density lipoprotein cholesterol ratio; eGFR, estimated glomerular filtration rate; FBG, fasting blood glucose; HDL-C, high–density lipoprotein cholesterol; hs–CRP, high–sensitivity C-reactive protein; LDL-C, low–density lipoprotein cholesterol; SBP, systolic blood pressure; TC, total cholesterol; UA, uric acid; UHR_2006,_ uric acid to high-density lipoprotein cholesterol ratio in 2006. | | | | | | |

| **Supplementary Table S3.** Hazard ratios for new-onset chronic kidney disease according to cumulative UHR quartile stratified by sex | | | | | | |
| --- | --- | --- | --- | --- | --- | --- |
|  |  | Quartile 1  HR (95% CI) | Quartile 2  HR (95% CI) | Quartile 3  HR (95% CI) | Quartile 4  HR (95% CI) | P for interaction |
| **Sex** |  |  |  |  |  | 0.047 |
| Male | Model 2 | Reference | 1.03 (0.93–1.15) | 1.16 (1.05–1.28) | 1.42 (1.28-1.56) |  |
| Female | Model 2 | Reference | 1.31 (1.13–1.52) | 1.37 (1.17–1.61) | 1.57 (1.29–1.91) |  |
| Model 2: adjusted for age, sex, smoking, alcohol consumption, education level, physical activity, body mass index, low-density lipoprotein cholesterol, estimated glomerular filtration rate, high sensitivity-C-reactive protein, diabetes, antidiabetic treatment, hypertension, and antihypertensive treatment. Abbreviations: CI, confidence interval; HR, hazard ratio. | | | | | | |

| **Supplementary Table S4.** Hazard ratios for new-onset CKD, low eGFR, and proteinuria in study participants without CKD according to CumUHR quartile (time-varying cox) | | | | | |  |
| --- | --- | --- | --- | --- | --- | --- |
|  | Quartile 1  HR (95% CI) | Quartile 2  HR (95% CI) | Quartile 3  HR (95% CI) | Quartile 4  HR (95% CI) | Per SD | |
| Participants, n | 9363 | 8649 | 9432 | 9445 |  | |
| CKD |  |  |  |  |  | |
| Model 1 | Reference | 1.16 (1.09–1.23) | 1.27 (1.19–1.34) | 1.53 (1.44–1.62) |  | |
| Model 2 | Reference | 1.14 (1.07–1.21) | 1.25 (1.17–1.32) | 1.48 (1.40–1.58) | 1.10 (1.09–1.11) | |
| Model 3 | Reference | 1.15 (1.08–1.22) | 1.27 (1.19–1.35) | 1.53(1.43–1.65) |  | |
|  |  |  |  |  |  | |
| Low eGFR |  |  |  |  |  | |
| Model 1 | Reference | 1.26 (1.15–1.38) | 1.37 (1.26–1.50) | 1.81 (1.66–1.98) |  | |
| Model 2 | Reference | 1.24 (1.14–1.36) | 1.38 (1.26–1.50) | 1.82 (1.67–1.99) | 1.11 (1.09–1.12) | |
| Model 3 | Reference | 1.26 (1.15–1.38) | 1.40 (1.28–1.54) | 1.90 (1.71–2.10) |  | |
|  |  |  |  |  |  | |
| Proteinuria |  |  |  |  |  | |
| Model 1 | Reference | 1.08 (1.00–1.16) | 1.21 (1.12–1.31) | 1.34 (1.24–1.45) |  | |
| Model 2 | Reference | 1.05 (0.97–1.14) | 1.17 (1.08–1.26) | 1.26 (1.17–1.37) | 1.08 (1.06–1.10) | |
| Model 3 | Reference | 1.06 (0.97–1.14) | 1.17 (1.08–1.27) | 1.28 (1.16–1.40) |  | |
| Notes: CKD was defined as eGFR <60 mL/min/1.73 m^2^ and/or proteinuria. Low eGFR was defined as eGFR <60 mL/min/1.73 m^2^. Proteinuria was defined as urinary protein ≥1+ on dipstick testing.  Model 1 was adjusted for age and sex.  Model 2 was adjusted for age, sex, smoking, alcohol consumption, education level, physical activity, body mass index, low-density lipoprotein cholesterol, high-sensitivity C-reactive protein, diabetes, antidiabetic treatment, hypertension, and antihypertensive treatment.  Model 3 was adjusted for all the variables in model 2 and UHR_2006._  Abbreviations: CI, confidence interval; CKD, chronic kidney disease; CumUHR, cumulative uric acid to high-density lipoprotein cholesterol ratio; eGFR, estimated glomerular filtration rate; HR, hazard ratio; SD, standard deviation; UHR_2006,_ uric acid to high-density lipoprotein cholesterol ratio in 2006. | | | | | |  |

| **Supplementary Table S5.** Hazard ratios for decline in eGFR and increase in proteinuria in study participants with CKD by CumUHR quartile (time-varying cox) | | | | | |  |
| --- | --- | --- | --- | --- | --- | --- |
|  | Quartile 1  HR (95% CI) | Quartile 2  HR (95% CI) | Quartile 3  HR (95% CI) | Quartile 4  HR (95% CI) | Per SD | |
| Participants, n | 3117 | 3830 | 3045 | 3033 |  | |
| Decline in eGFR category |  |  |  |  |  | |
| Model 1 | Reference | 1.04 (0.94–1.16) | 1.25 (1.12–1.39) | 1.59 (1.43–1.78) |  | |
| Model 2 | Reference | 1.05 (0.94–1.16) | 1.22 (1.09–1.36) | 1.53 (1.37–1.71) | 1.10 (1.07–1.13) | |
| Model 3 | Reference | 1.03 (0.93–1.15) | 1.18 (1.06–1.32) | 1.43 (1.26–1.61) |  | |
|  |  |  |  |  |  | |
| >30% decline in eGFR |  |  |  |  |  | |
| Model 1 | Reference | 1.06 (0.94–1.20) | 1.30 (1.15–1.48) | 1.65 (1.46–1.87) |  | |
| Model 2 | Reference | 1.07 (0.95–1.21) | 1.27 (1.12–1.44) | 1.57 (1.39–1.78) | 1.07 (1.04–1.09) | |
| Model 3 | Reference | 1.05 (0.93–1.18) | 1.22 (1.07–1.38) | 1.44 (1.25–1.64) |  | |
|  |  |  |  |  |  | |
| Increase in proteinuria |  |  |  |  |  | |
| Model 1 | Reference | 1.07 (0.95–1.19) | 1.19 (1.06–1.34) | 1.20 (1.06–1.34) |  | |
| Model 2 | Reference | 1.06 (0.95–1.19) | 1.16 (1.03–1.31) | 1.16 (1.02–1.32) | 1.04 (1.01–1.08) | |
| Model 3 | Reference | 1.08 (0.96–1.21) | 1.20 (1.06–1.37) | 1.25 (1.07–1.47) |  | |
| Notes: Decline in eGFR category was defined as a certain decrease in eGFR category accompanied by a ≥25% decrease in eGFR from baseline. A 30% decline in eGFR was defined as a >30% decrease in eGFR from baseline. An increase in proteinuria was defined as a certain increase in semiquantitative urinary protein (–, ±, 1+, 2+, 3+) on dipstick testing.  Model 1: adjusted for age and sex  Model 2 was adjusted for age, sex, smoking, alcohol consumption, education level, physical activity, body mass index, low-density lipoprotein cholesterol, high-sensitivity C-reactive protein, diabetes, antidiabetic treatment, hypertension, and antihypertensive treatment.  Model 3 was adjusted for all the variables in model 2 and UHR_2006._  Abbreviations: CI, confidence interval; CKD, chronic kidney disease; CumUHR, cumulative uric acid to high-density lipoprotein cholesterol ratio; eGFR, estimated glomerular filtration rate; HR, hazard ratio; SD, standard deviation; UHR_2006,_ uric acid to high-density lipoprotein cholesterol ratio in 2006. | | | | | |  |
